# Campbell Title Registrations to Date – September 2025, and Discontinued Protocols

**DOI:** 10.1002/cl2.70067

**Published:** 2025-09-16

##  

Details of new titles for systematic reviews or evidence and gap maps that have been accepted by the Editor of a Campbell Coordinating Group are published in each issue of the journal. If you would like to receive a copy of the approved title registration form, please send an email to the Managing Editor of the relevant Coordinating Group.

A list of discontinued protocols appears below these new titles. If you are interested to continue a project, please get in touch with the Managing Editor of the relevant Coordinating Group or email info@campbellcollaboration.org.

## 1 Ageing

The Impact of Technology Designed to Enhance IADL Management in Older Adults With Neurodegenerative Conditions on Informal Caregiver Wellbeing: A Systematic Review

Kevin Gines, Alyssa Weakley, Sarah Tomaszewski, Beth Tweedy, Bruce Abbott, Kaila Labrador, Rachel Park

14 August 2025

Interactions in Dementia Therapies: A Systematic Review

Ivy Meihua Su, Winsy Wing Sze Wong, Keyu Li

30 June 2025

Emerging Roles and Care Impacts of Geriatric Pharmacists in Outpatient Multidisciplinary Frailty and Geriatric Syndrome Management: A Systematic Review

Ravi Shankar, Fiona Devi, Xu Qian

14 August 2025

Physical Activity and Quality of Life in Older Adults With Chronic Illness and Disability: A Scoping Review

Wei Wei, Huaijin Xu, Xiaotian Gao, J. J. Pionke, Chungyi Chiu

14 August 2025

Mapping Financial Literacy Programs for the Ageing Population: Protocol for a Scoping Review Populations

Carina Sofia Teixeira Fernandes, Isabel Silva, Inês Gomes

2 September 2025

## 2 Business and Management

The Impact of Chatbot Customer Service in the E‐Commerce Industry on User Satisfaction: A Systematic Review

Xuemei Zhao, Zhifei Sun, Yijie Zhang, Liping Guo, Yufeng Wen, Wenjie Zhou

3 July 2025

Dual‐Career Academic Couples: A Scoping Review of Drivers, Benefits and Challenges

Blandine Ribotta, Antonia Velicu, Peter Hilpert, Bruno Lemaitre

20 June 2024

The Causal Impacts of Digital Training for Entrepreneurs in Developing Countries

Alejandro Estefan, Paul Winters

5 June 2025

## 3 Children and Young Persons Wellbeing

The Use of Digital Platforms to Enhance Adolescents' Sexual Reproductive Health Literacy in the Southern African Development Community: A Scoping Review

Olubunmi Ogbodu, Ayobami Adekola

22 June 2025

Global Incidence and Prevalence of Paediatric Acute‐Onset Neuropsychiatric Syndrome (PANS) and Paediatric Autoimmune Neuropsychiatric Disorder Associated With Streptococcal Infection (PANDAS) Diagnostic Label in Children Under 18 Years Old. A Systematic Review

Blessing Aina, Alastair Sutcliffe, Kate Green, Yan Lu

22 June 2025

Later‐Life Consequences of Firearm Violence Exposure in Childhood and Adolescence: A Systematic Review

Diego A. Diaz‐Faes, Charles Branas, Sonali Rajan

22 June 2025

Understanding Resilience in School Bullying Among Generation Y and Z: A Scoping Review of Elementary and Secondary Education

Hongyang Liu, Jana Kvintova, Lucie Vachova, Jiří Kantor

13 August 2025

Assessing Problematic Social Media Use: A Scoping Review Protocol Based on the COSMIN Methodology and TARES Statement

Amanda Cardoso, Matheus Rodrigues, Helen Mavichian, Victor Formigoni, Jessica Maruyama, Alexandre Serpa

13 August 2025

Determinants of Health Considerations in Normative Documents on the Management of Obesity in Children and Adolescents: A Systematic Review

Mirna Amaya, Kaloyan Kamenov, Lucero Lopez‐Perez, Ricardo Martinez, Omar Dewidar, Elizabeth Centeno Tablante, Gerardo Zamora, Marian Flaxman, Lindsay Bahureksa, Samantha Huey, Saurabh Mehta, Juan Peña‐Rosas

13 August 2025

The Relationship Between Partner Support and Breastfeeding Self‐Efficacy: The Protocol for a Global Mixed‐Methods Integrated Systematic Review

Eman Sharara, Rana Rizk, Bahia Abdallah, Rachael Eastham, Mark Limmer

22 June 2025

## 4 Climate Solutions

Predictive Factors for Enhancing Social‐Ecological Resilience Among Farmers Under Climate Change: A Systematic Review

Putri Setyowati, Maria Rola‐Rubzen, Ram Pandit, Emanuel Gomez

14 August 2025

## 5 Disability

Determinants of Weight Gain in Forensic Psychiatric Care: A Systematic Review and Meta‐Analysis

Justine Anthony, Joseph Lloyd Davies, Maria Goodwin

27 June 2025

## 6 Education

Compassion Fatigue in Medical Students and Junior Doctors: A Scoping Review Protocol

Jane Graves, Caroline Joyce, Moin Ahmed, Iman Hegazi

4 August 2025

Educational Burnout and Mental Disorders Among School and University Students: Systematic Review Protocol

Klaudia Bochniarz, Paweł Jurek, Paweł Atroszko

4 August 2025

Use of Real‐Time Visual Feedback Technologies in Singing Students in Educational Settings: A Scoping Review

Glòria Vila Aymerich, Miquel Alsina

4 August 2025

Exploring the Use of Simulation to Teach Clinicians Spirituality: A Mixed Methods Systematic Review

Lorraine Henshaw, Adam Boughey, Jessica Runacres, Wilfred McSherry, Joseph Natalello

20 May 2025

Improving Progression in Public Primary Schools in Low‐ and Middle‐Income Countries: A Systematic Review of Available Options

Perez Kirya, Peter Kasadha, Francis Kizito, Ally Kinyaga, Rhona Mijumbi‐Deve, Rose Izizinga, Edward Kayongo

20 May 2025

The State of the Literature Regarding Anti‐DEI Policy in Higher Education Settings: A Scoping Review

Helen Yates, Arthur Frankel, David Conley, Jenneffer Sixkiller, Morgan Stone

6 June 2025

## 7 International Development

Reusable Pads Versus Disposable Pads For Menstrual Hygiene Management for Women and Girls of Reproductive Age Globally

Nain Yuh, Charlotte Ndum, Damaris Ntam, Clarisse Chenwi, Ernest Alang, Patrick Okwen

14 July 2025

## 8 Knowledge Translation and Implementation

The Current Practice and Opportunities of Information Security for Telehealth in Public Hospital: Systematic Review

Tagel Mallese, Mniyichel Kasa

14 August 2025

Interventions to Improve Recruitment and Retention of Mental Health Professionals: A Systematic Review

Narendar Manohar, Hiroko Fujimoto, Jeff Looi, Paul Maguire, Stephen Kisley, Tarun Bastiampillai, Stephen Robson, Samuel Harvey, Peter Baldwin

15 July 2025

Utilisation of Genetics and Genomics in Primary Care: An Evidence and Gap Map

Braulio Mark Valencia Arroyo, Shona Bates, Jialing Lin, Rafal Chomik, Peter Brown, Chris Dietz, Limin Mao, Michael Kidd

3 July 2025

Leadership Development Program Outcomes for Women: A Systematic Review and Meta‐Analysis

Daisy Morris, Kathleen Riach, Sana Rauf, Helena Teede

3 July 2025

Effectiveness and Implementation of Antimicrobial Stewardship (AMS) Interventions for Reducing Antimicrobial Resistance (AMR) and Improving Clinical Outcomes in Global Healthcare Settings: A Mixed‐Method Approach

R. Rajalakshmi, Jayaraj Mymbilly Balakrishnan, Sreedharan Nair, Sohil Khan, Mohammed Salim, Uday Kumar R., Sneh Shalini, N. V. Vajid, Girish Thunga

15 July 2025

## 9 Methods

Use of GRADE in Campbell Systematic Reviews: A Cross‐Sectional Survey

Xuping Song, Rui Wang, Yunze Han, Qiyin Luo, Jing Tang, Xinye Guo, Yan Ma, Yue Hu, Xufei Luo, Yaolong Chen, Kehu Yang, Howard White, Vivian Welch

22 June 2025

Prognostic Models for Joint Longitudinal Variables and Recurrent Events: A Systematic Review

Thomas Spain, Maria Sudell, Laura Bonnett

20 June 2025

## 10 Social Welfare

Opioid Agonist Therapy for Fentanyl‐Related Opioid Use Disorder: Protocol for a Systematic Review

Austin Solak, Jack Boynton, J. Jacob Riches, Allison Souter, Kathryn Dong, Maryam Zaree, Nicolas Woods, Alla Iansavichene, Christopher Byrne

17 June 2025

Understanding the Health Care Experiences of Women Who Use Illegal Substances: An Intersectional Scoping Review

Marjan Kekanovich, Tyler Glass, Amanda Ross‐White, Danielle Macdonald, Susan Bartels, Jacqueline Galica

18 May 2025

Early Post‐Trauma Programmes for Preventing the Development of Trauma Reactions and Reducing Psychological Distress in Employees Exposed to Work‐Related Potentially Traumatic Events: A Systematic Review

Trine Filges, Malene Kildemoes, Elizabeth Bengtsen

11 June 2025

